# Author Correction: Type-printable photodetector arrays for multichannel meta-infrared imaging

**DOI:** 10.1038/s41467-024-49948-w

**Published:** 2024-07-04

**Authors:** Junxiong Guo, Shuyi Gu, Lin Lin, Yu Liu, Ji Cai, Hongyi Cai, Yu Tian, Yuelin Zhang, Qinghua Zhang, Ze Liu, Yafei Zhang, Xiaosheng Zhang, Yuan Lin, Wen Huang, Lin Gu, Jinxing Zhang

**Affiliations:** 1 School of Electronic Information and Electrical Engineering, Institute of Advanced Study, Chengdu University, Chengdu, 610106 China; 2 School of Integrated Circuit Science and Engineering, National Exemplary School of Microelectronics, University of Electronic Science and Technology of China, Chengdu, 610054 China; 3 School of Integrated Circuits, Tsinghua University, Beijing, 100084 China; 4 College of Integrated Circuit Science and Engineering, National and Local Joint Engineering Laboratory for RF Integration and Micro-Packing Technologies, Nanjing University of Posts and Telecommunications, Nanjing, 210023 China; 5 School of Physics and Astronomy, Beijing Normal University, Beijing, 100875 China; 6 Key Laboratory of Multiscale Spin Physics, Ministry of Education, Beijing, 100875 China; 7 Institute of Physics, Chinese Academy of Science, Beijing National Laboratory of Condensed Matter Physics, Beijing, 100190 China; 8 School of Materials and Energy, University of Electronic Science and Technology of China, Chengdu, 610054 China; 9 School of Materials Science and Engineering, Tsinghua University, Beijing, 100084 China

**Keywords:** Nanophotonics and plasmonics, Electrical and electronic engineering

Correction to: *Nature Communications* 10.1038/s41467-024-49592-4, published online 18 June 2024

The original version of this Article contained a few errors in the author affiliations and in the Acknowledgements section.

Affiliation 5 was incorrectly named as “Department of Physics, Beijing Normal University, Beijing 100875, China” instead of “School of Physics and Astronomy, Beijing Normal University, Beijing, 100875, China”. Moreover, a new affiliation number 6, “Key Laboratory of Multiscale Spin Physics, Ministry of Education, Beijing, 100875, China”, has now been added for the following authors: Yu Tian, Yuelin Zhang and Jinxing Zhang. The other affiliation numbers have been corrected accordingly.

In the Acknowledgements section, the National Natural Science Foundation of China Grant No. 52225205 and the National Key Research and Development Program of China Grant No. 2021YFA0718700 were missing. Moreover, the contribution of the Fundamental Research Funds for the Central Universities was erroneously omitted.

This has been corrected in the PDF and HTML versions of the Article.

