# Supplementary material for: Type-printable photodetector arrays for multichannel meta-infrared imaging
Source: Nat Commun. 2024 Jun 18;15:5193. doi: 10.1038/s41467-024-49592-4 (PMC11189553; doi:10.1038/s41467-024-49592-4)
Supplement: Supplementary file 1 — Supplementary Information [file 41467_2024_49592_MOESM1_ESM.pdf]

Supplementary Information

Type-printable photodetector arrays for multichannel meta-infrared imaging

Junxiong Guo[1,2,#,*], Shuyi Gu[1,#], Lin Lin[2,#], Yu Liu[3,4*], Ji Cai[1], Hongyi Cai[1], Yu Tian[5], Yuelin Zhang[5], Qinghua Zhang[6], Ze Liu[1], Yafei Zhang[1], Xiaosheng Zhang[2], Yuan Lin[7], Wen Huang[2,*], Lin Gu[8], and Jinxing Zhang[5,*]

[1] School of Electronic Information and Electrical Engineering, Institute of Advanced Study, Chengdu University, Chengdu 610106, China

[2] School of Integrated Circuit Science and Engineering, National Exemplary School of Microelectronics, University of Electronic Science and Technology of China, Chengdu 610054, China

[3] School of Integrated Circuits, Tsinghua University, Beijing 100084, China

[4] College of Integrated Circuit Science and Engineering, National and Local Joint Engineering Laboratory for RF Integration and Micro-Packing Technologies, Nanjing University of Posts and Telecommunications, Nanjing 210023, China

[5] Department of Physics, Beijing Normal University, Beijing 100875, China

[6] Institute of Physics, Chinese Academy of Science, Beijing National Laboratory of Condensed Matter Physics, Beijing 100190, China

[7] School of Materials and Energy, University of Electronic Science and Technology of China, Chengdu 610054, China

[8] School of Materials Science and Engineering, Tsinghua University, Beijing 100084, China

[#] These authors contributed equally: Junxiong Guo, Shuyi Gu, and Lin Lin

* Corresponding authors

E-mail addresses: guojunxiong@cdu.edu.cn (J.X. Guo), y-liu-17@tsinghua.org.cn (Y. Liu), uestchw@uestc.edu.cn (W. Huang), and jxzhang@bnu.edu.cn (J.X. Zhang)

Contents

Supplementary Note 1. Estimation of graphene Fermi level 3

Supplementary Note 2. Numerical calculation for graphene dynamical conductivity 4

Supplementary Note 3. Finite element method (FEM) for electric field simulation 4

Supplementary Note 4. Regulating resonance frequency by rescaling the ferroelectric domain width 5

Supplementary Note 5. Infrared imaging simulation 6

Supplementary Note 6. Neural network training and recognition 7

Supplementary Figure 1. Schematic of infrared imaging. 8

Supplementary Figure 2. Structure analysis of ferroelectric superdomain doping of graphene. 9

Supplementary Figure 3. Optical image of fabricated device array. 11

Supplementary Figure 4. Schematic of experimental setup. 12

Supplementary Figure 5. Transmission extinction characterization. 13

Supplementary Figure 6. Photocurrent characterization. 14

Supplementary Figure 7. Key parameters characterization of fabricated photodetector. 15

Supplementary Figure 8. Schematic of infrared imaging workflow herein. 16

Supplementary Figure 9. Comparison of infrared imaging workflow. 17

Supplementary Figure 10. Mapping the objective temperature using device response. 18

Supplementary Figure 11. Comparison of infrared imaging using single-channel and multichannel photodetector array. 19

Supplementary References 20

Supplementary Note 1. Estimation of graphene Fermi level

In this work, the graphene Fermi level was estimated by the analytical model derived as follows[1]. The electronic band of graphene near the Dirac point **K** can be described as:

$$E(\mathbf{k}, \pi) = \hbar v_F k \tag{S1}$$

and

$$E(\mathbf{k}, \pi) = -\hbar v_F k \tag{S2}$$

where **k**+**K** is the momentum of the Dirac Fermions, v_F is the Fermi velocity of $\sim 1.1\times10^6$ m s^{-1}. According to the G phonon pattern of graphene, the band structure can be derived from the modified equation:

$$E(\mathbf{k}, \pi^*/\pi, \mathbf{u}) = \pm\hbar v_F|\mathbf{k}-\mathbf{s}(\mathbf{u})| \tag{S3}$$

where $\boldsymbol{s}$ is a vector of Dirac point shifted from K, $s = u\sqrt{2\langle D_\Gamma^2\rangle_F}$ and $\mathbf{s}\cdot\mathbf{k} = 0$, $\langle D_\Gamma^2\rangle_F = 45.6$ eV^2 Å^{-2}, Γ is the charged particle scattering rate. When a phonon exists, the phonon energy $\hbar\omega_{EF}$ can be determined as:

$$\hbar\Delta\omega = \hbar\omega_{E_F} - \hbar\omega_0 = \frac{\hbar}{2M\omega_0}\frac{d^2\Delta E}{(du)^2} \tag{S4}$$

where E_F is the graphene Fermi energy, M is the atomic (carbon) mass, ω_0 is the frequency in undoped state, $\Delta\omega << \omega_0$, and ΔE is the variation of the electronic energy. In graphene, the G-peak position is dependent of its Fermi level, according to the experiments which breakdowns the adiabatic Born–Oppenheimer approximation, can be expressed as:

$$\Delta E(u) = \frac{4A}{(2\pi^2)}\int_{E(\mathbf{k},\pi^*,\mathbf{0})<E_F} E(\mathbf{k}, \pi^*, \mathbf{u})d^2k + O(u^3) \tag{S5}$$

where A is the unit cell area. Combining the Equations S3-S5, it can be carried out the Fermi level E_F as a function of the frequency shifts as:

$$\hbar\Delta\omega = \frac{\hbar A\langle D_\Gamma^2\rangle_F}{\pi M\omega_0(\hbar v_F)^2}|E_F| = \alpha'|E_F| \tag{S6}$$

where $\alpha' = 4.39\times10^{-3}$.

Supplementary Note 2. Numerical calculation for graphene dynamical conductivity

The complex conductivity of graphene σ_g can be calculated within the random-phase approximation (RPA), the dynamic optical response of graphene can be derived from Kubo formula in a complex form consisting of interband and intraband contribution[2,3], shown in Equation S7.

$$\sigma_{\mathrm{g}} = \sigma_{\mathrm{intra}} + \sigma_{\mathrm{inter,1}} + i\sigma_{\mathrm{inter,2}} \tag{S7}$$

Here, the intraband conductivity of graphene σ_g follows the Drude-like model, shown in Equations S8 and S9.

$$\sigma_{\mathrm{intra}} = \sigma_0 \frac{4\mu_{\mathrm{c}}}{\pi} \frac{1}{\hbar\tau_1 - i\hbar\omega} \tag{S8}$$

$$\sigma_0 = \frac{\pi e^2}{2h} \tag{S9}$$

where τ_1 is the relaxation rate associated with intraband transitions, and μ_c is the chemical potential of graphene, equals to the Fermi level of graphene E_F. The interband conductivity of graphene $\sigma_{\mathrm{inter,1}}$ and $\sigma_{\mathrm{inter,2}}$ follows the form of Equations S10 and S11, respectively.

$$\sigma_{\mathrm{inter,1}} = \sigma_0 \left(1 + \frac{1}{\pi} \arctan \frac{\hbar\omega - 2\mu_{\mathrm{c}}}{\hbar\tau_2} - \frac{1}{\pi} \arctan \frac{\hbar\omega + 2\mu_{\mathrm{c}}}{\hbar\tau_2} \right) \tag{S10}$$

$$\sigma_{\mathrm{inter,2}} = -\sigma_0 \frac{1}{2\pi} \ln \frac{(2\mu_{\mathrm{c}} + \hbar\omega)^2 + \hbar^2\tau_2^2}{(2\mu_{\mathrm{c}} - \hbar\omega)^2 + \hbar^2\tau_2^2} \tag{S11}$$

where τ_2 is the relaxation rate associated with interband transitions.

Supplementary Note 3. Finite element method (FEM) for electric field simulation

For our designed photodetector consisting of graphene and periodically polarized BFO strips, the near-field distribution at plasmonic resonance frequency with different Fermi levels and domain periods were simulated using FEM. Graphene was modeled using a uniaxial anisotropic permittivity assuming that the graphene layer was a surface current with non-thickness. In this model, the graphene conductivity was described using the Drude-like model (as seen in Supplementary Note 2 for details). The thickness of BFO was 25 nm, corresponding to the actual epitaxial growth film. The refractive index of BFO was using a model of Kumar et al. Periodical conditions were imposed on the double sides of our simulated region. The width of our simulated region corresponds to the

experimental polarization domain width, that is the simulated width varying from 100 to 500 nm; The graphene Fermi level was using the estimated results via the Raman G-peak shits. The E_F of graphene on upward and downward domains was set as +121 meV and –448 meV, respectively, according to the Raman-G band frequencies shift.

Supplementary Note 4. Regulating resonance frequency by rescaling the ferroelectric domain width

In the nonretarded regime of $q << q_0$, the dielectric-graphene-dielectric architecture behaves a TM plasmon mode, and the graphene plasmon wave vector q follows Equation S12,

$$q \approx \frac{i2\omega\varepsilon_0\varepsilon_r}{\sigma(q,\omega)} \tag{S12}$$

where $q_0 = \omega/c$ is the incident light wave vector in free space, ω, c ε_0, and ε_r are the anglar frequency of incident light, vaccum light speed, vaccum dielectric constant, relative dielectric constant of BFO, respectively[4]. $\sigma(q, \omega)$ is the dynamical conductivity of graphene, as well defined as σ in Equation S7 of Supplementary Note 2. For our designed device, because of the long-wavelength incident light and high-doping of graphene printed by BFO, the graphene conductivity is dominated by intraband contributions, following Equations S8 and S9. We submit Equations S8 and S9 into Equation S12 to achieving the graphene plasmon dispertion relation in long-wavelength region[5], as shown Equation S13.

$$q(\omega) = \frac{2\pi\varepsilon_0\varepsilon_r}{e^2E_F}\omega^2\left(1+\frac{i}{\tau_1\omega}\right) \tag{S13}$$

To resonantly enhance the light-matter interactions, the wavevector difference between incident light wave and excited surface plasmon wave must be compensated by the periodic grating condition as Equation S14,

$$\mathrm{Re}\{q(\omega)\} = q_0\sin\theta \pm N\frac{2\pi}{a} \tag{S14}$$

where θ is the incident angle, N = 1, 2, 3, …, respectively, $\frac{2\pi}{a}$ represents the reciprocal vector of the grating, and a is the period of grating[6]. Therefore, we can achive the graphene plasmon resonace disperation relation as Equation S15,

$$\omega_{\mathrm{spr}} = \left(\frac{e^2 E_{\mathrm{F}}}{2\pi\hbar^2 \varepsilon_0 \varepsilon_{\mathrm{r}}} \left(q_0 \pm N \frac{\pi}{d} \right) \right)^{\frac{1}{2}} \tag{S15}$$

where a = 2d, and d is the ferroelectric domain width. For the given Fermi levels of ferroelectric superdomain printing graphene, we can easily regulated the resonance frequency by rescaling the ferroelectric domain width.

Supplementary Note 5. Infrared imaging simulation

The infrared imaging simulation is established to reproduce a complete physical imaging process using two types of photodetector array. As shown in Supplementary Fig. 8a, the process consists of three sections, including target temperature collection, data proceesing, deep training and recognition. The target temperature were captured using a commercial camera (Melexis, MLX90640). The measured temperature response of the commercial device and corresponded fitting result according to the datasheet in manual is shown in Supplementary Fig. 10. The environment temperature remains 20°C when we collect the target temperatures.

The data processing is conducted by using an open source project called OPENZYNQ (https://github.com/openzynqhardware/openzynq). In brief, the OPENZYNQ project uses a ZYNQ7010/7020 BGA400 pin 4-layer PCB. The core board configuration mainly includes: (1) 16-bit DDR3, (2) PS and PL reset buttons, one each, (3) QSPI W25Q64/128, (4) SD card slot, (5) CH340 serial to USB converter, (6) JTAG interface, (7) USB dedicated I/O port, (8) PL with a 50MHz active crystal oscillator and PS with a 33.3 MHz active crystal oscillator, and (9) automatic boot mode switching: SD card boot when an SD card is inserted, QSPI boot when not inserted. It is worth noting that, to ensure fairness in subsequent image recognition, the total number of optoelectronic detectors simulated for infrared imaging is the same in both approaches. The difference lies in the fact that in the traditional single-channel array (SCA) photodetectors, one device corresponds to one pixel, while in the multi-channel array (MCA) photodetector, 6 devices form a single pixel (see Fig. 1b in main text for details). In other words, the MCA image has one-sixth the number of pixels compared to SCA, and the infrared imaging comparison of core workflow between traditional SCA and MCA herein is shown in Supplementary Figs. 8 and 9.

Moreover, according to the selective responsivity feature in our fabricated MCA detectors

(Supplementary Figs. 5 to 7), we quantified the objective temperature using a simple Lorentz method given as Equation S16,

$$y = f(x) = (1-\alpha)\frac{A}{B^2 + (x - x_0)^2} \tag{S16}$$

where y, and x represent response amplitude, and input temperature, respectively, x_0 is the central point of Lorentz function, A and B are constants related the selective responses of fabricated device, and α is a tunable parameter for modulating the response amplitude. As shown in Supplementary Fig. 10, it demonstrates the typical calculated response as a function of normalized temperature and their corresponding merged response characteristics. The detail code of the Lorentz fitting method employing in this work has attached on an open-source website (https://github.com/We1wu/Multichannel-meta-infrared-imaging, DOI: 10.5281/zenodo.11544077).

Supplementary Note 6. Neural network training and recognition

Supplementary Figure 8b shows the artificial neural network we employed in this work. Back-propagation (BP) algorithm is used for training and recognition processes. The activation function in Hidden layer and Output layer is ReLU. The learning rate is set as a fix value 0.01. As mentioned in Supplementary Note 5, the input images size of 144×192 pixels and 72×64 pixels in each channel, convolution layers of $5\times5\times20$ and $3\times3\times12$ for the structure of neural network within SCA and MCA-based models, respectively. The classification outputs are set as (0, 1, 2, 3, 4, 5), and (0, 1)in recognition of gesture shapes, and curled fingers, respectively.

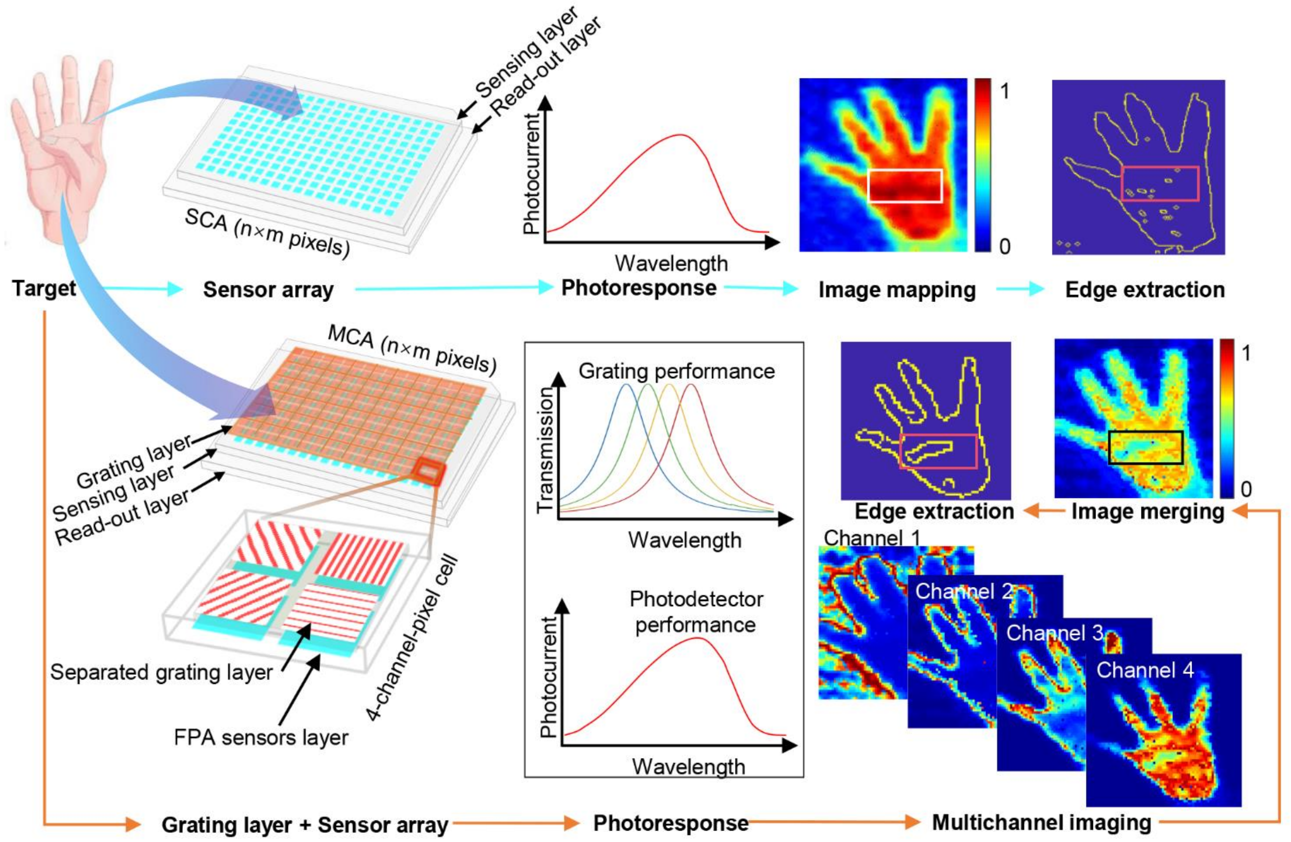

Supplementary Figure 1. Schematic of infrared imaging.

A comparison between the traditional infrared imaging approach using single-channel array (SCA) photodetectors (top panel) and the multi-channel array (MCA) photodetectors by combining focal plane array (FPA) sensors with separated gratings (bottom panel). The MCA detectors-based infrared imager accurately recognize a curled thumb, whereas the conventional SCA approach cannot.

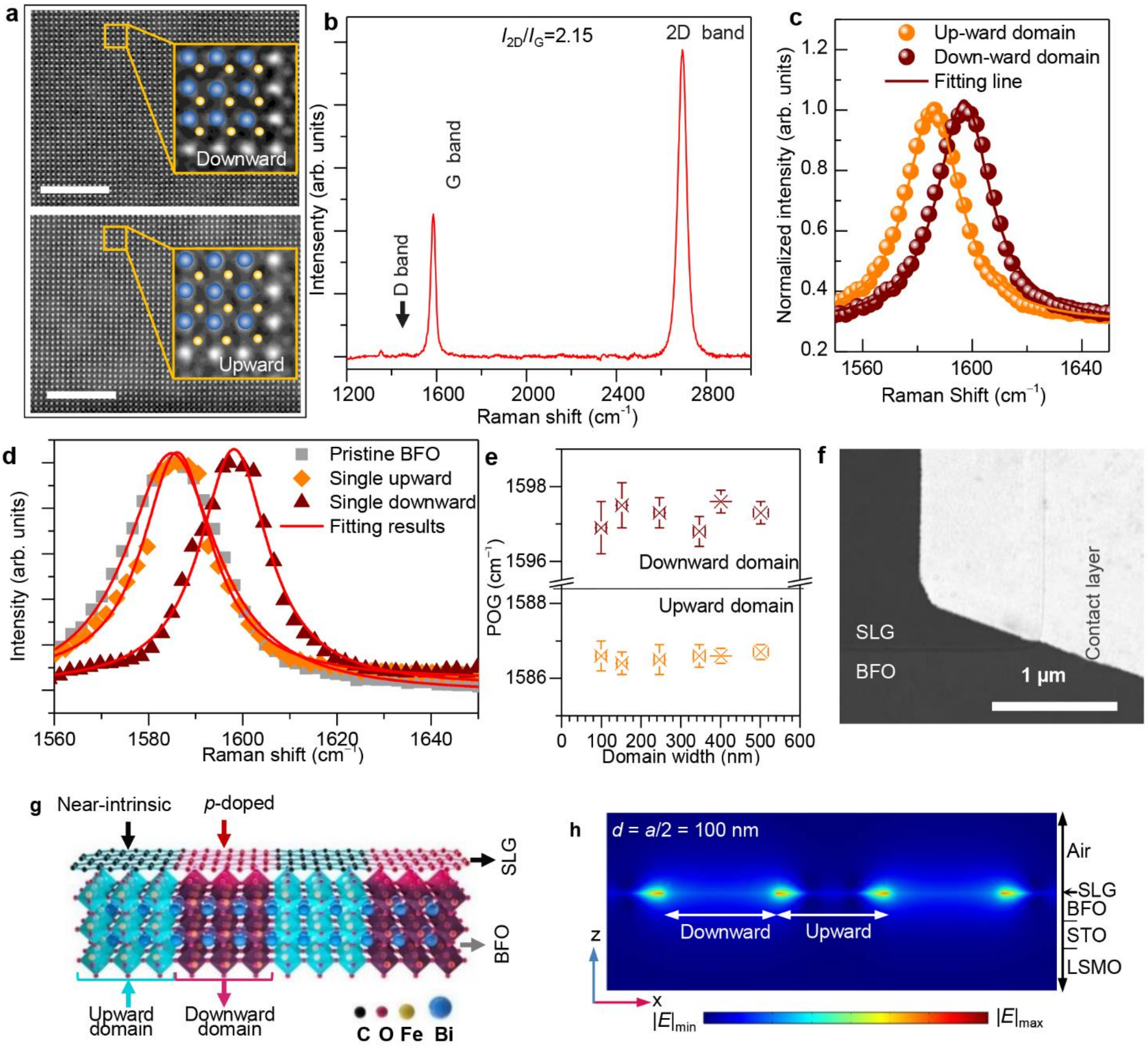

Supplementary Figure 2. Structure analysis of ferroelectric superdomain doping of graphene. (**a**) Cross-sectional view of transmission electron microscopy images of the upward and downward domains in BFO thin film, with a scale bar of 5 nm. (**b**) Raman spectrum of single-layer graphene on unpolarized BFO film. Distinct by a high 2D-to-G peak intensity ratio (I_{2D}/I_G = 2.15) and a low D band intensity, suggesting low defect density. G band positioned at 1583.5 cm^{-1} and 2D band at 2694.6 cm^{-1} indicate near-intrinsic graphene characteristics[7-9]. (**c**) Averaged Raman frequencies for graphene on upward and downward domains, with solid lines representing the Lorentz fitting results. (d) Average Raman G-band frequences for graphene on fresh BFO (gray squares), single upward domain (orange diamonds), and single downward domain (wine triangles), measured at 1585.3 cm^{-1}, 1586.1 cm^{-1}, and 1597.2 cm^{-1}, respectively. Solid lines depict Lorentzian fit outcomes, confirming

that graphene on upward domain retains near-intrinsic properties similar to the pristine BFO. (**e**) Position of graphene G-band (POG) peaks as a function of BFO superdomain period. The data are simultaneously extracted from the AFM-Raman mapping in Fig. 3b of main text. The POG peaks for graphene on upward and downward domains were around 1586 and 1597 cm^{-1}, respectively, confirming the feasibility of spatial printing of graphene carrier density without patterning of graphene sheet. The error bars indicate standard deviation. (**f**) Raw SEM image of graphene/BFO-based photodetector. (**g**) Schematic of the device focusing on the graphene/BFO interface, illustrating the p-doped and near-intrinsic graphene behaviors on downward and upward domains, respectively. (**h**) Simulated electric field intensity for graphene plasmon resonator by a two-dimensional model. It shows that the highly confined graphene plasmons are located in the borders of upward/downward domains at the graphene/BFO interface. SLG denotes single-layer graphene.

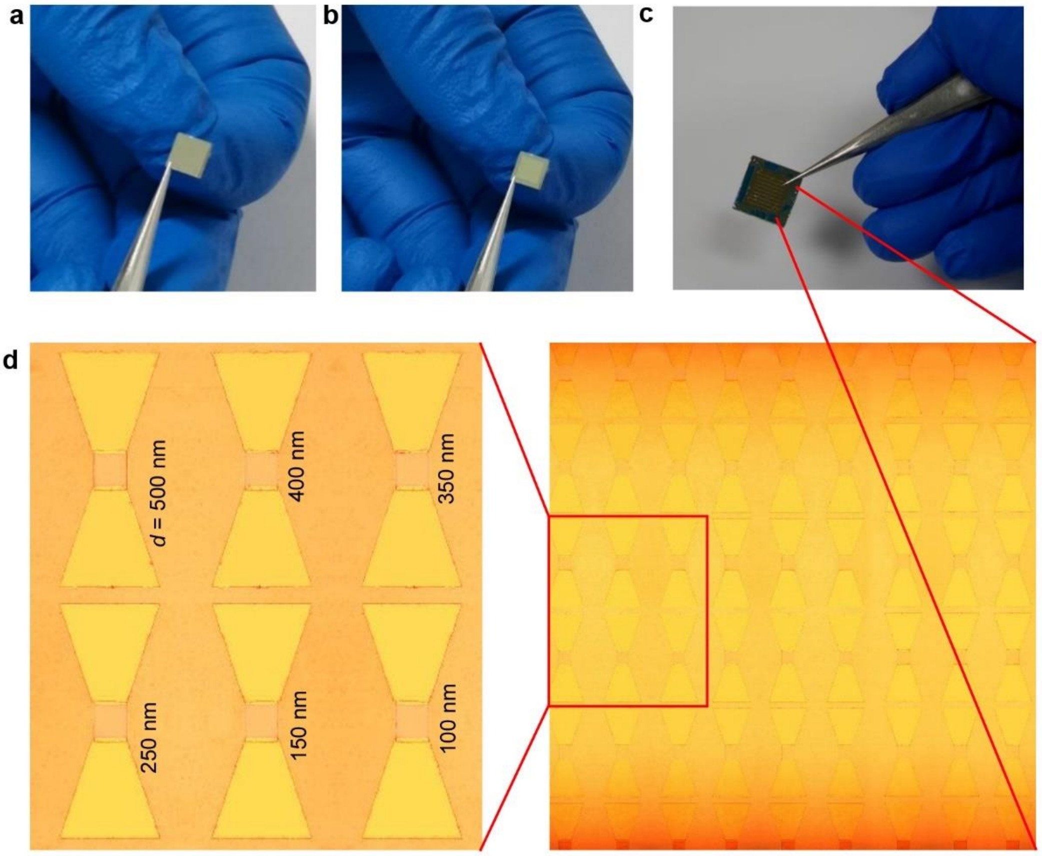

Supplementary Figure 3. Optical image of fabricated device array.

(**a**) Bare BFO film epitaxially grown on an STO substrate with an LSMO coating layer. (**b**) Completed device of transferred graphene onto BFO film. Sample dimensions in (**a**) and (**b**) are 5 mm × 5 mm designed for optical measurements, including Raman, transmission performances. (**c**) The assembled photodetector array featuring graphene/BFO active layers complemented by Ti/Pd/Au contact layers. The sample size in (**c**) is 10 mm × 10 mm, which accommodates additional photodetector cells to facilitate photoelectric performance assessments. (**d**) Optical microscopy image of our fabricated photodetector array (6×9 devices, 3×3 cells, right panel) and specific enlarged view of single cell (2 ×3 devices, left panel).

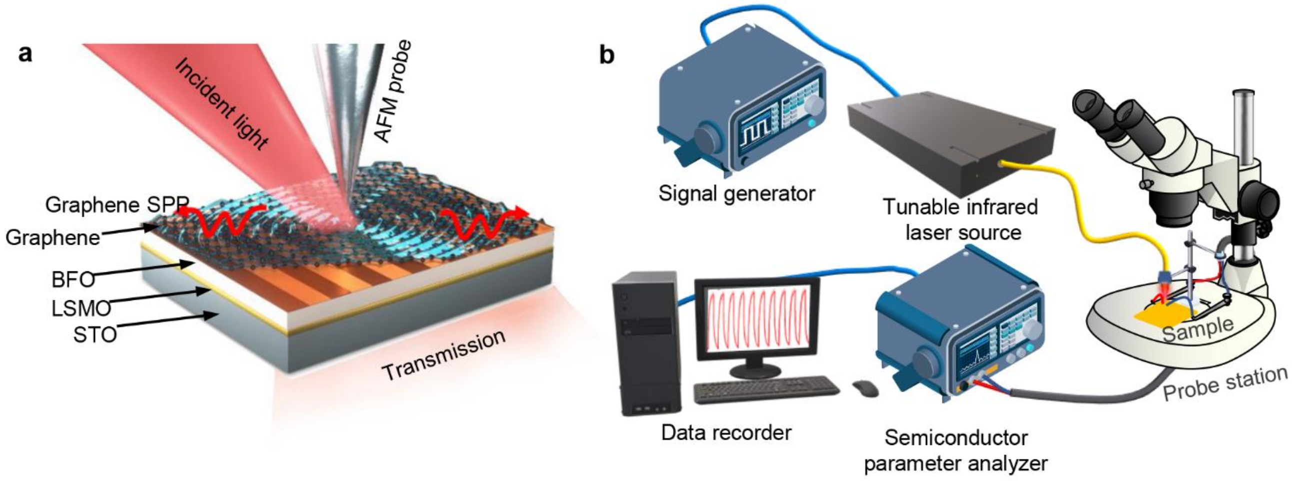

Supplementary Figure 4. Schematic of experimental setup.

(**a**) Scheme of infrared transmission microscopy measurement. The AFM probe is used to guide the incident infrared light into specific zone of device. (**b**) Workflow of photocurrent characterization in fabricated device. The laser wavelengths are modulated from 2.3 to 10 µm with a spot size of 5 mm, covering the entire device.

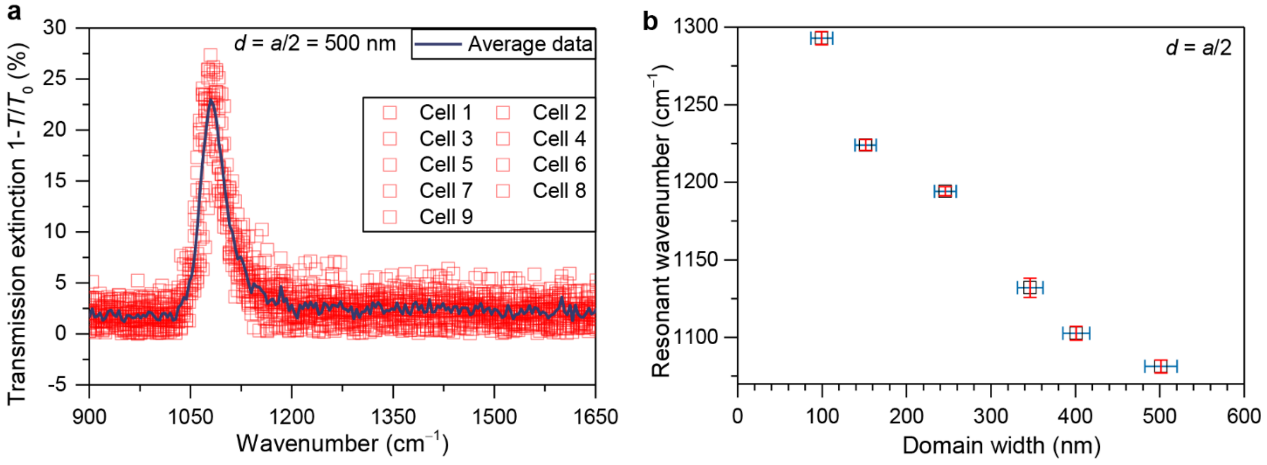

Supplementary Figure 5. Transmission extinction characterization.

(**a**) Measured transmission extinction spectra of the graphene/BFO photodetector with a same domain width of 500 nm. The solid hollow squares and solid line are the experimental results and averaged data, respectively, extracted from 9 cells. (**b**) Spectral response characterization using resonant wavenumber as a function of ferroelectric domain width. The error bars indicate standard deviation.

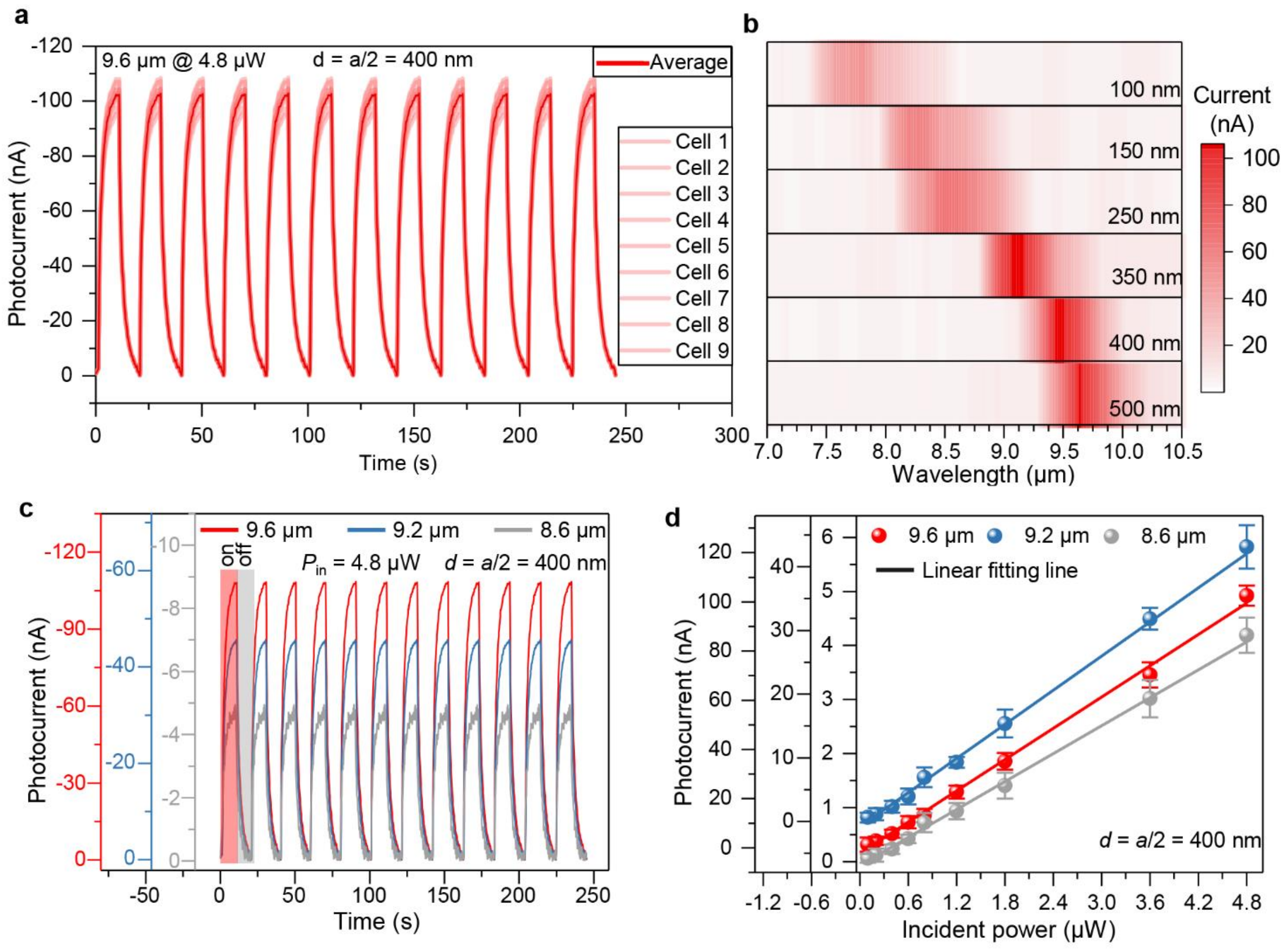

Supplementary Figure 6. Photocurrent characterization.

(**a**) Measured *I*–*t* characteristics of the graphene/BFO photodetectors with a same ferroelectric domain width of 400 nm. (**b**) Highest photocurrent as function of incident wavelength depending on the width of the BFO superdomain. (**c**) Measured *I*–*t* characteristics of the graphene/BFO photodetector under different laser wavelengths. The light red and gray strips represent the on and off states of incident lasers. (**d**) Photocurrent as function of incident power. The solid lines are the liear fitting results. The data are extracted from the highest values of *I*-*t* curves in each device. The error bars represent the standard deviation. The currents of the device were collected measured under zero source–drain bias voltage. The photocurrents were recorded within 9 devices shown in Supplementary Fig. 3d.

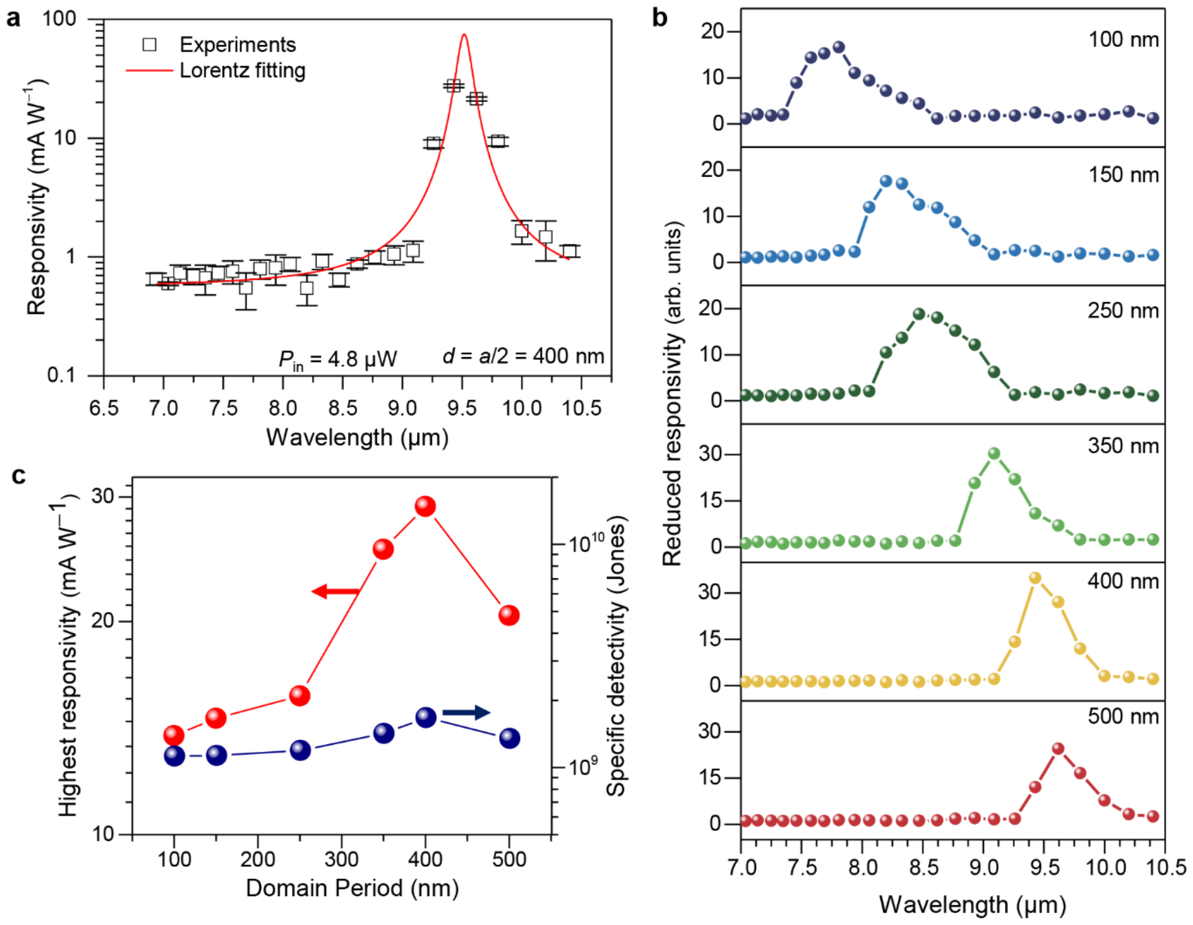

Supplementary Figure 7. Key parameters characterization of fabricated photodetector.

(**a**) Measured responsivity of the graphene/BFO photodetectors with a same ferroelectric domain width. The solid line is the fitting result using a simple Lorentz method. The error bars represent the standard deviation. (**b**) Reduced responsivity (R_{max}/R_{min}) as functions of incident wavelength depending on the width of the BFO superdomain. (c) Highest responsivity and specific detectivity as function of incident wavelength depending on the width of the BFO superdomain. The data were collected measured under zero source–drain bias voltage within nine devices shown in Supplementary Fig. 3d.

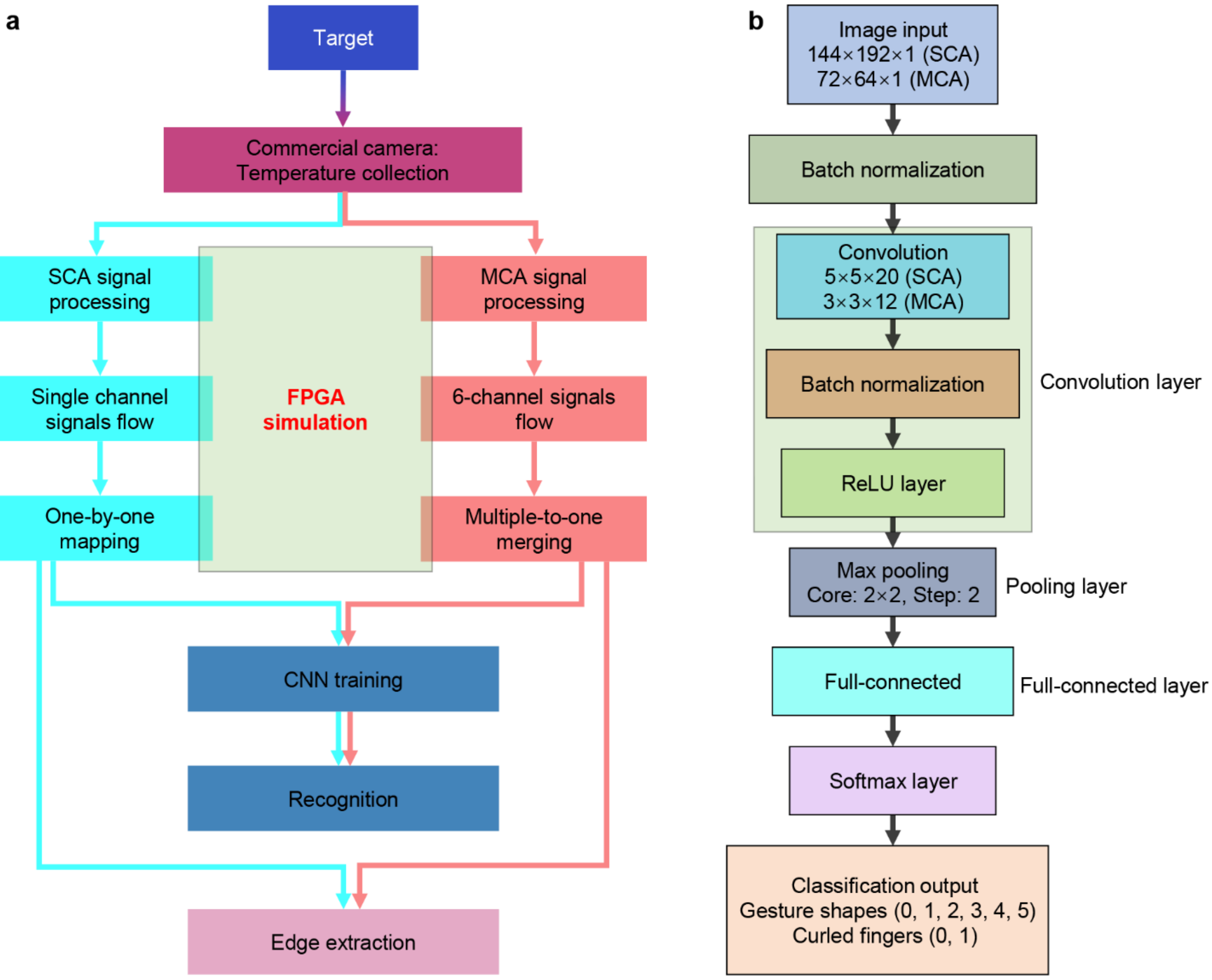

Supplementary Figure 8. Schematic of infrared imaging workflow herein.

(**a**) Overall process for deeping imaging. (**b**) Deep learning and recognition process.

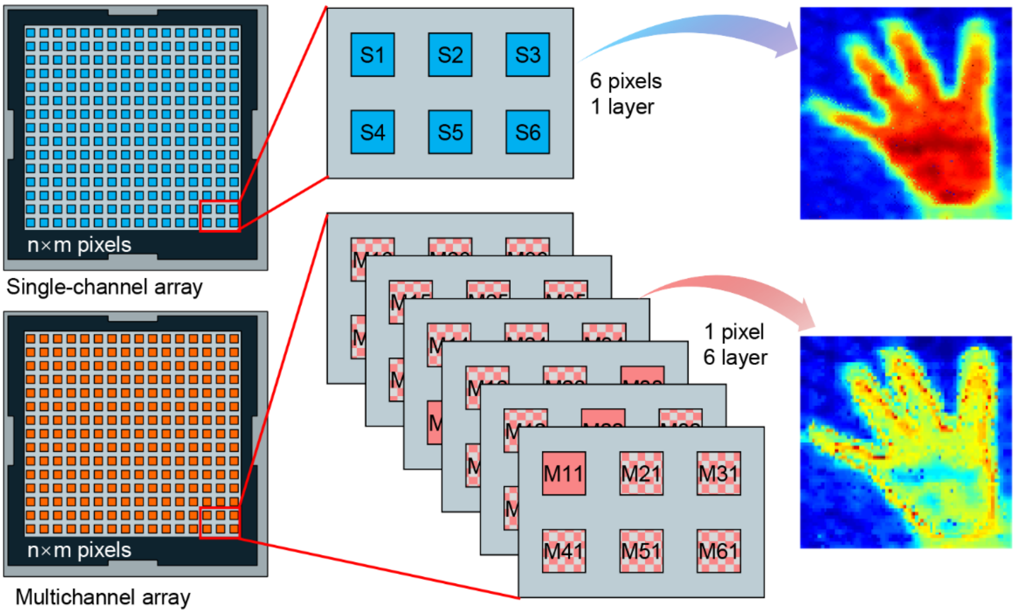

Supplementary Figure 9. Comparison of infrared imaging workflow.

Standard infrared imaging technique using single-channel detector array (top panel) and proposed meta-infrared imaging approach using multichannel plasmonic detector array herein (bottom panel). The right columns are typical simulations of gesture-4 infrared imaging.

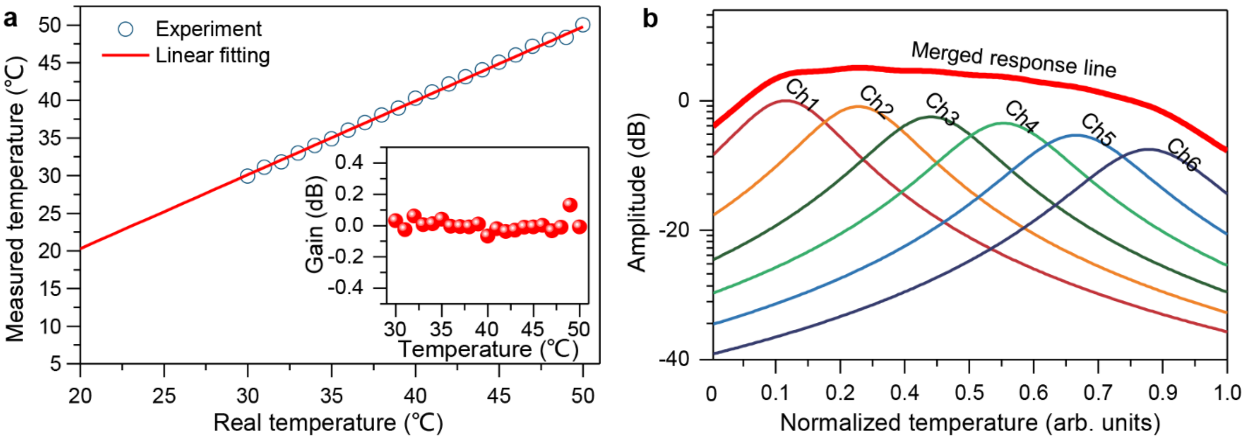

Supplementary Figure 10. Mapping the objective temperature using device response.

(**a**) Measured temperature as a function of objective temperature and corresponded fitting result by using commercial manual. The inset is the corresponded gain by comparing measured temperature and fitted temperature. (**b**) Calculated response amplitude as a function of normalized temperature using Lorentz method.

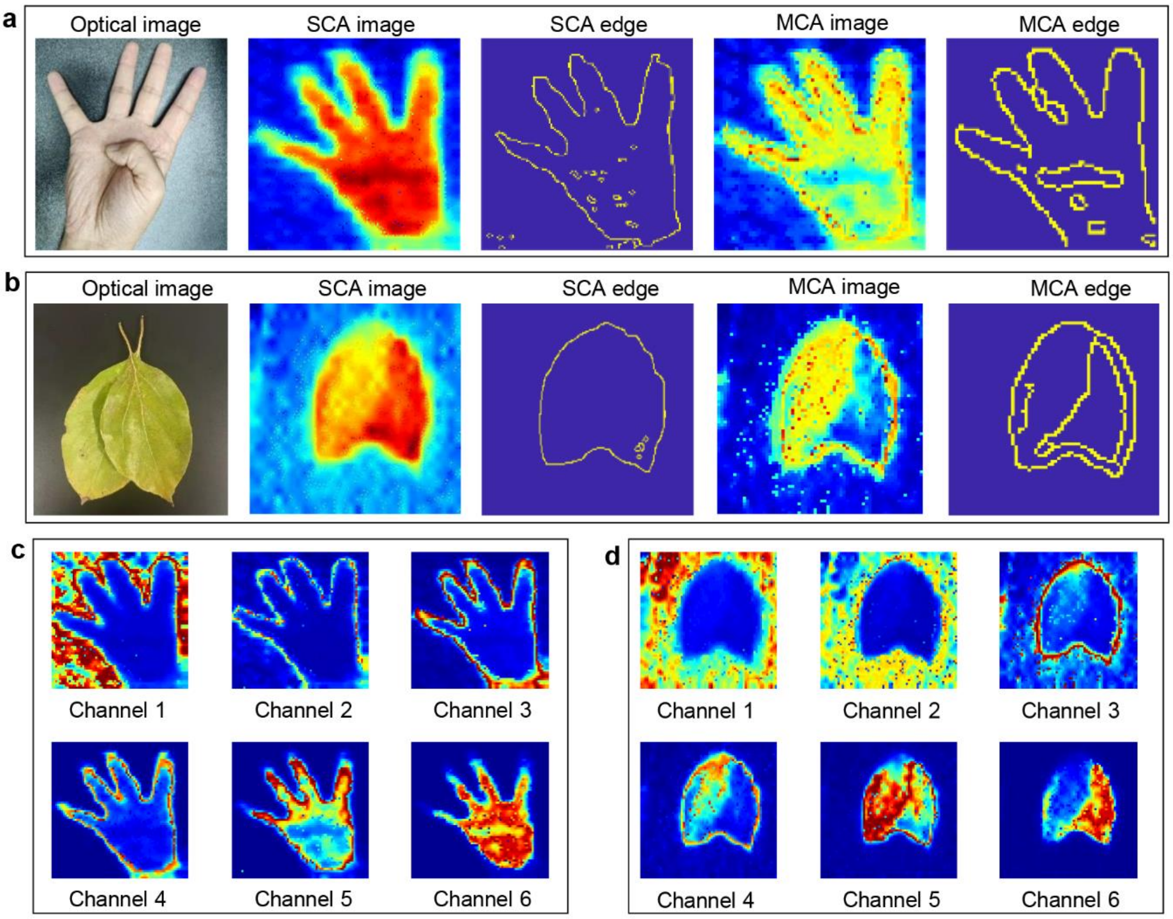

Supplementary Figure 11. Comparison of infrared imaging using single-channel and multichannel photodetector array.

Standard optical image, infrared snapshots alongside associated edge profiles for gesture 4 with curled thumb finger (**a**) and partially covered leaves (**b**) using both single- and multichannel photodetector arrays. Channel-by-channel simulation results using the proposed 6-channel model for Meta-infrared imaging of curled gesture (**c**) and overlapped leaves (**d**).

Supplementary References

1. Pisana, S. et al. Breakdown of the adiabatic Born-Oppenheimer approximation in graphene. *Nat. Mater.* **6**, 198-201 (2007).
2. Hwang, E. H., Das Sarma, S. Dielectric function, screening, and plasmons in two-dimensional graphene. *Phys. Rev. B.* **75**, 6 (2007).
3. Stauber, T. Plasmonics in Dirac systems: from graphene to topological insulators. *J. Phys. Condens. Mat.* **26**, 123201 (2014).
4. Barnes, W. L., Dereux, A., Ebbesen, T. W. Surface plasmon subwavelength optics. *Nature* **424**, 824-830 (2003).
5. Low, T., Avouris, P. Graphene plasmonics for terahertz to mid-infrared applications. *Nano Lett.* **8**, 1086-11101 (2014).
6. Maier, S.A. *Plasmonics: fundamentals and applications*, Springer Science & Business Media (2007).
7. Ferrari, A.C., Basko D.M. Raman spectroscopy as a versatile tool for studying the properties of graphene. *Nat. Nanotechnol.* **8**, 235-246 (2013).
8. Ferrari, A.C., et al. Raman spectrum of graphene and graphene layers. *Phys. Rev. Lett.* **97**, 187401 (2006).
9. Das, A., et al. Monitoring dopants by Raman scattering in an electrochemically top-gated graphene transistor. *Nat. Nanotechnol.* **3**, 210-215 (2008).
